# Production and characterisation of activated carbon and carbon nanotubes from potato peel waste and their application in heavy metal removal.

**DOI:** 10.1007/s11356-019-06594-w

**Published:** 2019-11-20

**Authors:** Ahmed I. Osman, Jacob Blewitt, Jehad K. Abu-Dahrieh, Charlie Farrell, Ala’a H. Al-Muhtaseb, John Harrison, David W. Rooney

**Affiliations:** 1 School of Chemistry and Chemical Engineering, Queen’s University Belfast, David Keir Building, Stranmillis Road, Belfast, BT9 5AG Northern Ireland; 2 Chemistry Department, Faculty of Science-Qena, South Valley University, Qena, 83523 Egypt; 3 South West College, Cookstown, Co., Tyrone, BT80 8DN Northern Ireland, UK; 4 School of Mechanical and Aerospace Engineering, Queen’s University Belfast, Belfast, BT9 5AH Northern Ireland, UK; 5 Department of Petroleum and Chemical Engineering, College of Engineering, Sultan Qaboos University, Muscat, Oman

**Keywords:** Potato peel waste, Biomass, Activated carbon, Carbon nanotubes, Pyrolysis, Multi-wall carbon nanotubes

## Abstract

Herein, activated carbon (AC) and carbon nanotubes (CNTs) were synthesised from potato peel waste (PPW). Different ACs were synthesised via two activation steps: firstly, with phosphoric acid (designated PP) and then using potassium hydroxide (designated PK). The AC produced after the two activation steps showed a surface area as high as 833 m^2^ g^−1^ with a pore volume of 0.44 cm^3^ g^−1^, where the raw material of PPW showed a surface area < 4 m^2^ g^−1^. This can help aid and facilitate the concept of the circular economy by effectively up-cycling and valorising waste lignocellulosic biomass such as potato peel waste to high surface area AC and subsequently, multi-walled carbon nanotubes (MWCNTs). Consequently, MWCNTs were prepared from the produced AC by mixing it with the nitrogen-based material melamine and iron precursor, iron (III) oxalate hexahydrate. This produced hydrophilic multi-wall carbon nanotubes (MWCNTs) with a water contact angle of θ = 14.97 °. Both AC and CNT materials were used in heavy metal removal (HMR) where the maximum lead absorption was observed for sample PK with a 84% removal capacity after the first hour of testing. This result signifies that the synthesis of these up-cycled materials can have applications in areas such as wastewater treatment or other conventional AC/CNT end uses with a rapid cycle time in a two-fold approach to improve the eco-friendly synthesis of such value-added products and the circular economy from a significant waste stream, i.e., PPW.

**Electronic supplementary material:**

The online version of this article (10.1007/s11356-019-06594-w) contains supplementary material, which is available to authorized users.

**Graphical abstract:**
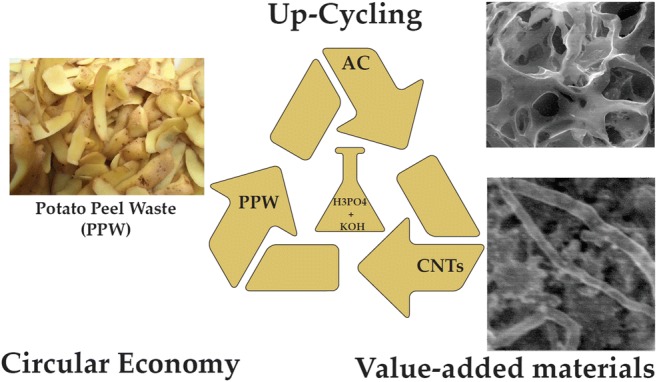
.

## Introduction

Currently, there is a global reliance on fossil fuel sources derived from crude oil or coal-based briquettes directly used as fuel. This reliance brings with it the environmental concerns that are often attributed to products produced via these means, such as CO_2_ emissions. Therefore, study into alternative sources and methods to produce energy is crucial (Kim et al. 2019, Vuppaladadiyam et al. 2019, Yasar et al. 2001, Zhang et al. 2019). One such alternative source stems from the utilisation of biomass to make products suited for energy applications. There are many benefits for the use of biomass, which is desirable when considering international agreements such as the Paris agreement on climate change as it provides additional incentive for using biomass-based sources in energy applications. Biomass has the benefit of being carbon negative in its production, giving the possibility of carbon neutrality over a product’s lifecycle to be obtained, whilst also proving to be a sustainable product throughout its lifespan. However, there are many challenges of using biomass as a fuel due to its low energy density, along with the high moisture content which leads to an increase in transportation costs per unit of energy contained in the fuel (Mašek et al. 2008). There are several studies on other ways of utilising biomass. For example, anaerobic digestion which involves the conversion of biomass to biogas, gasification which converts biomass into hydrogen-based gases and pyrolysis which can produce a wide range of products. The pyrolysis products vary from bio-oils with potential fuel applications or use as a chemical feedstock, gases with high heating values and char which has uses directly as fuel or as a feedstock. One of these feedstocks is the production of activated carbon (AC), which in turn can provide one potential pathway towards the production of carbon nanotubes (CNTs) via the pyrolysis route which is the underlying focus of this particular study.

Pyrolysis is a process which involves heating matter in an inert atmosphere to produce useful products, with the product properties depending upon various factors (Adam et al. 2013, Lago et al. 2018, Mašek et al. 2009, Yang et al. 2019). One of which is the chemical composition of the original biomass. Herein, potato peel waste is to be considered as a feedstock for this study due to its wide availability and presence in most demographic regions of the world. Globally, the total potato production is estimated at over 376 million tonnes per year, and whilst much of this is sold to consumers directly, others go into use in the production of additional foodstuffs such as crisps or chips. Within these sectors, potato peel is often overlooked as a priority product, providing a useful resource which can be exploited for use or the manufacture of useful products. Potatoes are a versatile crop that are grown on six of the seven continents with the exception of Antarctica. This suggests that its use in the manner proposed could be utilised all over the world with relative ease. In Northern Ireland, within the local demographic of Ireland, potatoes account for around 36% of the total sale output of field crops at around £24 million.

Pyrolysis of biomass claims a wide range of potential use-case applications (Sonoyama et al. 2006). However, despite the use of bio-oil from pyrolysis having potential to be used as a transportation fuel, it is not the most practical choice of product from the pyrolysis process (Yu et al. 2015). This is due to its lacking economical feasibility to replace existing methodologies of making conventional transportation fuel (Bridgwater 1999). Therefore, research into the best way to utilise the products of pyrolysis and the particular product that fits this criterion should be considered.

One major product obtained via pyrolysis is AC, which is a porous carbon that can be used for the adsorption of both inorganic and organic compounds (Marsh & Reinoso 2006). The production of AC involves the activation of biomass, which can be achieved via physical activation or chemical activation (Wang et al. 2019). Physical activation involves carbonisation followed by high-temperature pyrolysis in the temperature range of 800–1000 °C. This is considered an energy-intensive method due to the high temperatures required for the production route. Chemical activation also involves pyrolysis. However, in this case, the biomass is first activated with chemical agents such as H_3_PO_4_, ZnCl_2_ or KOH. The pyrolysis of the activated biomass then occurs at much lower temperatures than that of the physical activation, typically at temperatures of approximately 500 °C.

The three most common activating agents are ZnCl_2_, H_3_PO_4_ and KOH; high surface area (> 2000 m^2^ g^−1^) AC was prepared using ZnCl_2_, also a micropore AC (620 m^2^ g^−1^) was prepared using 40 wt% ZnCl_2_ impregnation (Moreno-Piraján & Giraldo 2011, Yorgun et al. 2009). However, ZnCl_2_ is considered as the most expensive activating agent, while the cheapest activating agent is H_3_PO_4_ and it has been extensively used in the literature (Kyzas et al. 2016b, Nahil & Williams 2012, Romero-Anaya et al. 2011, Yorgun et al. 2009). Gómez-Tamayo et al. studied the production of ACs via different concentrations of phosphoric acid and pyrolysis temperatures; it was found that 60% H_3_PO_4_ concentration along with pyrolysis temperature of 450 °C produced AC with a surface area of 1723 m^2^ g^−1^ (del Mar Gómez-Tamayo et al. 2008). Heidari et al. prepared ACs with high surface area (2595 m^2^ g^−1^) using H_3_PO_4_ as a first activating agent followed by KOH as a second activating agent (Heidari et al. 2014). It was mentioned that a two-step activation method produces up to 25% higher carbon yields activated carbon than that of a single-step activation method along with 50% higher surface area and pore volume also (Ravichandran et al. 2018).

Although chemical activation of biomass includes the above steps throughout the literature (Arampatzidou & Deliyanni 2016, Kalderis et al. 2008, Kyzas & Deliyanni 2015, Kyzas et al. 2016a), additional steps can be applied to improve the porosity of the final product. Both acid and base washing are common methods used alongside the activation. The washing of biomass can act to remove metal ion components which, if present, compromise and lower the yield of the desired products. The acid washing after the pyrolysis of activated biomass can act to remove any excess activating agent. Some studies wash with a base, activate and then wash with acid (Liou 2010). Whereas, others forgo the initial wash favouring just an acid wash after the pyrolysis (Le Van & Luong Thi 2014, Muniandy et al. 2014, Williams & Reed 2006).

Further processing of ACs can also produce CNTs, which were first discovered in 1991. They are a form of carbon similar to Buckminsterfullerene (C_60_), containing an array of hexagonally connected carbon atoms. However, the atoms do not fold down on themselves forming a tube structure as opposed to a ball-like structure seen with Buckminsterfullerene. They have huge potential due to their high mechanical strength, thermal and electrical conductivity (Baughman 2002). CNTs can be produced via numerous methodologies including, but not limited to, chemical vapour deposition (CVD), laser ablation and arc discharge. The production of CNTs from arc discharge involves a high purity graphite anode and cathode within a helium atmosphere where a voltage is applied to develop CNTs upon the graphite cathode. Laser ablation involves the vaporisation of graphite via exposure to a laser within a controlled atmosphere with temperatures of around 1200 °C. Both laser ablation and arc discharge share a significant flaw of not being well suited to scalability (Thostenson et al. 2001). CVD involves the decomposition of carbon-containing gases to produce carbon nanotubes. The CVD method also has had some commercial success to produce single-walled CNTs (SWCNTs) (Thostenson et al. 2001).

Due to the limitation of finite resources and the cost barrier of some of these sought after high-value materials, the application and thought of producing them from a renewable, common and regrowable crop such as potatoes provides added incentive to research the production of activated carbon and carbon nanotubes from waste lignocellulosic biomass to be able to facilitate industry and the environment’s needs. The additional enhanced material properties also help produce novel, undiscovered and unconventional materials which can be used to help the day to day lives of others and a wide range of industries and their needs worldwide.

There are limited instances within the literature that produces CNTs from the feedstock of AC with a nitrogen-based compound and either an iron or nickel-based compound and melamine before being pyrolysed twice at different temperatures (Yao et al. 2017a, Yao et al. 2017b). To the best of the authors’ knowledge, there is no study on producing AC via two-stage activation method from potato peel waste (PPW), then further processed to produce multiwall CNTs, where the produced materials are used for heavy metal removal. Herein, we prepared AC materials from the abundant PPW feedstock via two consecutive activation steps using H_3_PO_4_ and KOH. The produced AC was then used for the preparation of CNTs, followed by the application in heavy metal removal study. To the best of the authors’ knowledge, this is the first detailed study on converting PPW into value-added material and its application in wastewater treatment.

## Materials and methods

The chemicals used in the present study were all analytical grade, supplied by Sigma-Aldrich, UK, and were used without further purification. Orthophosphoric acid (85 wt% in H_2_O, 99.99% trace metals basis), hydrochloric acid (ACS reagent, 37%), potassium hydroxide (pellets, 99.99% trace metals basis), melamine (2,4,6-triamino-1,3,5-triazine, 99%), iron oxalate (Fe_2_(C_2_O_4_)_3_6H_2_O, ≥ 99%), lead acetate (99.99%) and methanol (HPLC grade, ≥ 99.9%, Sigma-Aldrich, UK) were all obtained from Sigma-Aldrich.

### PPW sample

Potato peel waste was used as the raw material in this study. The raw sample was oven-dried at around 100 °C for around 72 h. After drying, it was ground to a small particle size of less than 300 μm. Some sample was ground to a size of less than 106 μm, so the properties of the initial sample could be determined via testing.

#### Activated carbon preparation

The production of AC was carried out via two consecutive activation methods as follows:

##### First activation with phosphoric acid

Activated carbon was produced by mixing 11.4 g of potato peel waste with 11.9 mL 85% phosphoric acid (H_3_PO_4_) and 150 mL of deionised water. The mixture was stirred at 100 °C for 2 h before being left to dry for 24 h. The dried sample was then pyrolysed in a tube furnace using a fixed bed reactor at 500 °C with a 30 min hold time and a heating rate of 2 °C min^−1^. The pyrolysis was carried out under N_2_ atmosphere with a flow rate of 100 mL min^−1^. The sample was then cooled to room temperature before it was washed with water to remove any impurities that may have been gained during activation and to neutralise the pH to around 6–7. The sample was dried for 24 h at 120 °C. The sample was designated as PP.

##### Second activation with potassium hydroxide

The PP sample was subjected to a second activation, where 3.12 g of PP was mixed with KOH with a weight ratio of 1:3.5 and deionised water was added before being stirred at 100 °C for 1 h. The sample was then dried for 24 h at 120 °C. The dried sample was then pyrolysed at 500 °C, with a 30 min hold time at a heating rate of 2 °C min^−1^ under N_2_ atmosphere. The sample was then cooled to room temperature before being washed initially with HCl to remove any impurities and then deionised water to neutralise the pH. The sample was dried for 24 h at 120 °C. The sample was designated as PK.

#### Carbon nanotubes preparation

For the production of CNTs, 1 g of PK sample was mixed with 17.5 g of melamine and 0.5 g of iron oxalate precursor. It was then stirred for 4 h in a methanol solution before being dried at 120 °C for 24 h. The sample was then pyrolysed at 600 °C with a heating rate of 2 °C min^−1^ and held for 3 h before being subsequently pyrolysed to 900 °C with a heating rate of 2 °C min^−1^ and held for 1 h. The sample was subsequently cooled to room temperature before undergoing washing and drying for 24 h at 80 °C. The sample was designated as CNTs.

### Lignocellulosic biomass characterisation

Powder X-ray diffraction (XRD) was carried out using a PANalytical X’Pert Pro X-ray diffractometer. This diffractometer was equipped with a CuK_α_ X-ray source with a wavelength of 1.5405 Ǻ. The diffractograms were collected up to 2θ = 80 °. The X-ray tube was set at 40 kV and 40 mA.

Brunauer-Emmett-Teller (BET) analysis was performed using a Micromeritics ASAP 2020 system. BET surface area and pore volume were measured by N_2_ adsorption and desorption isotherms at liquid nitrogen temperature (− 196 °C).

Scanning electron microscopy (SEM) was carried out on a FEI Quanta 250 FEG MKII with a high-resolution environmental microscope (ESEM) using XT Microscope Control software and linked to an energy-dispersive X-ray (EDX) detector. Two types of detectors were used in SEM analysis, the Everhart-Thornley Detector (ETD) which is used to detect secondary electrons emitted from the sample and Back-Scattered Electron Detector (BSED). The EDX used was a 10 mm^2^ silicon drift detector (SDD)-x-act from Oxford Instruments which utilises Aztec® EDS analysis software. Both systems used the same chamber.

The static contact angle of the PPW and prepared CNTs with water was measured using a contact angle meter equipped with a CCD camera (FTA1000 Drop Shape Instrument-B Frame system).

The composition of the potato peel waste sample was characterised by means of proximate and ultimate analyses. Elemental (C, H and N) analysis was performed using a Perkin Elmer PE2400 CHNS/O Elemental Analyzer. The oxygen content was calculated by difference from the data obtained by the Perkin Elmer PE 2400 CHNS/O Elemental Analyzer machine.

Thermogravimetric analysis (TGA) technique was completed by heating the sample from 50–950 °C with different heating rates in a stream of N_2_ gas flowing at 40 cm^3^ min^−1^ using the Mettler Toledo Thermogravimetric analyser Pyris TGA/DSC. The heating rates employed were 2.5, 10, 20 and 30 °C min^−1^, respectively, for the analysis of PPW dry plant. However, an analysis was completed for the CNT product also with only one heating rate, 10 °C min^−1^.

The morphology of the activated carbon (PK) and CNTs' surface was characterised by transmission electron microscopy (TEM) (JEOL 2100 with high tension of 200 kV and a point resolution of 0.24 nm).

XPS spectra were obtained using multiprobe X-ray photoemission spectroscopy (XPS) (Omicron Nanotechnology, Germany) with a monochromatic Al Kα radiation (hν = 1486.6 eV) working at 15 kV, 20 mA. High-resolution XPS spectra were deconvoluted to individual components using Casa XPS software (Casa Software Ltd). The intrinsic carbon C 1 s peak at 284.6 eV was used as calibration. In order to avoid charging effect, sample surface was flooded with electron beam during measurement.

Fourier transform infrared (FT-IR) spectroscopy was operated using a PerkinElmer Spectrum, a beam splitter in the wavenumber range of 4000–400 cm^−1^.

### Batch adsorption test

The lead precursor was dissolved in deionised water to prepare the desired initial concentration (100 ppm). Heavy metal removal test was performed at a pH of ~ 6 with the liquid to solid (L/S) ratio of 100. A sample was taken after an hour then at consequent different time intervals of 1, 24, 72 and 168 h. Inductively coupled plasma optical emission spectrometry (ICP-OES) was used to determine the elemental analysis of the samples in the heavy metal absorption test. The solution of each sample was analysed with an ICP optical emission spectrometer (Optima 4300 DV, PerkinElmer).

## Results and Discussion

### Lignocellulosic biomass characterisation

#### XRD analysis

**Fig. 1:**
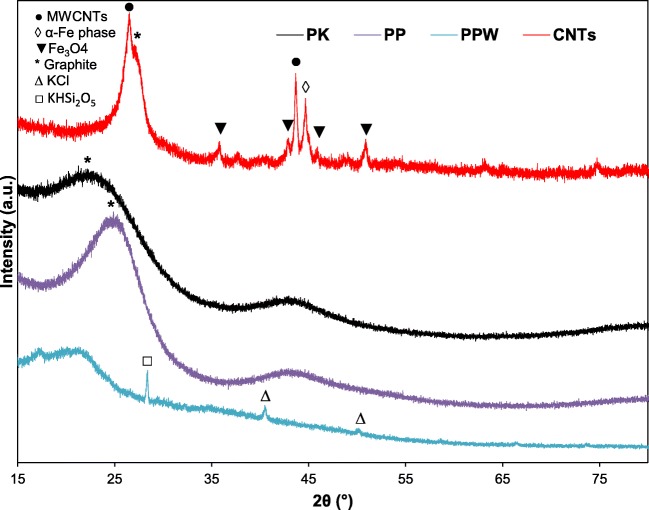
XRD patterns of raw PPW, activated carbon firstly using phosphoric acid (AC-P) and secondly using potassium hydroxide (KOH) along with the carbon nanotubes (CNTs) derived from potato peel waste samples

X-ray diffraction of PPW, PP, PK and CNTs are shown in Fig. 1. The raw PPW sample showed the diffraction lines that correspond to crystalline and amorphous cellulose phases at 2θ of 18 and 22°, respectively (JCPDS No 03-0226) (Nanda et al. 2012). The raw PPW also showed diffraction lines that corresponded to KHSi_2_O_5_ at 2θ of 28.4° (JCPDS No 19-968) along with KCl at 2θ of 40.6 and 50.2° (JCPDS No 01-073-0380) (Xue et al. 2015) (Swain & Bahadur 2013). For both the AC (PP and PK) samples, there are only two diffraction lines at 2θ of 24.9 and 42.9° for the PP and 22.3 and 43.1° for PK, which are attributed to crystalline graphite (JCPDS No 41-1487) with slightly different crystallinities (Wang et al. 2009). On the other hand, the CNTs sample showed more diffraction lines than the ACs samples, mainly in the form of the diffraction line at 2θ of 26.6°, corresponding to crystalline graphite. Three of the remaining diffraction lines correspond to Fe_3_O_4_ at 2θ of 35.9, 43.6 and, 50.6° (JCPDS No 19-0629) (Yu & Kwak 2010), where the diffraction line at 2θ of 44.7° corresponded to α-Fe phase (JCPDS No 06-0696) (Yao et al. 2017b) (Kramm et al. 2012). The CNTs sample also showed the two diffraction peaks which corresponded for the MWCNTs at 2θ of 26.6° (002 plane), which also could be observed in a hexagonal graphite along with the diffraction line at 2θ of 43.6° (100 plane) (JCPDS No 01-0646) (Güler 2014) (Moazzen et al. 2019).

#### S_BET_ analysis

**Fig. 2:**
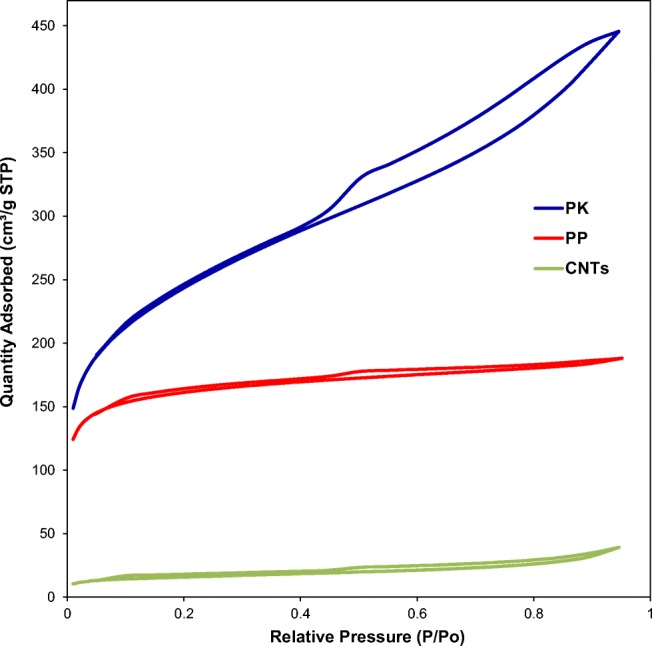
Nitrogen adsorption-desorption isotherms at 77 K of samples of potato peel waste activated carbon firstly using phosphoric acid (PP) and secondly using potassium hydroxide (PK) along with the carbon nanotubes (CNTs)

As can be seen in Table 1 and Fig. 2, there is a significant increase in surface area and pore volume between the PPW and PP samples. Initially, PPW showed lower surface of < 4 m^2^ g^−1^ with 0.002 cm^3^ g^−1^ pore volume. Both of these figures were significantly enhanced through the first activation stage using phosphoric acid (PP) to reach 676 m^2^ g^−1^ and 0.26 cm^3^ g^−1^, respectively. This suggests that the phosphoric acid activation created new pores within the structure. The adsorption/desorption isotherms in all samples are of IV type in the relative pressure range of 0.4–0.9, implying the development of mesoporous carbonaceous materials which is in line with the work of Morali et al. (2018). Clearly, there was a further increase in the S_BET_ and pore volume through the second activation process in PK sample with 833 m^2^ g^−1^ and 0.44 cm^3^ g^−1^, respectively, as shown in Table 1 along with Fig. 2. This improvement in the surface area measurements is ascribed to the removal of the oxygen, nitrogen and hydrogen content in the lignocellulosic biomass during the formation of mesoporous carbonaceous material (Danish & Ahmad 2018). Conversely, the CNTs sample has a much lower surface area and pore volume than those of ACs with 52 m^2^ g^−1^ and 0.05 cm^3^ g^−1^, respectively, as shown in Table 1 along with Fig. 2. The surface area of CNTs can vary widely depending on the wall thickness of the CNT. For instance, a single-wall CNT (SWCNT) could have a surface area of up to a theoretical maximum of 1375 m^2^ g^−1^. While, a surface area of 50, 175 and 500 m^2^ g^−1^ corresponds to a 40-walled, 10-walled and 3-walled CNTs, respectively (Birch et al. 2013a). Given the surface area herein, it is clear that the produced CNTs via this method are MWCNTs with a 40-wall thickness. The low surface area can also correspond to the presence of multilayer polygonal particles, impurities, amorphous carbon and large graphite platelets within the structure of the CNTs (Birch et al. 2013b, Zhu et al. 2003). It is worth noting that the formation of MWCNTs has advantages over SWCNTs, due to the enhanced thermal stability, low product cost per unit and chemical properties (Choi & Zhang n.d.).

**Table 1:**
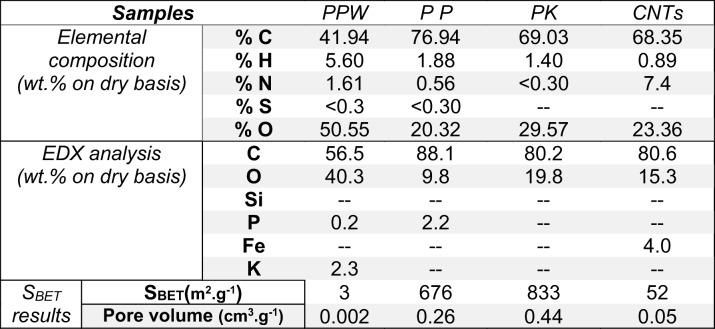
Physicochemical characterisations of potato peel waste (PPW) along with the ACs (PP, PK) and CNTs samples

The ultimate analysis of PPW showed a wt% of carbon, hydrogen, nitrogen and sulphur of 41.9, 5.6, 1.6 and < 0.3 wt%, respectively. The results are in agreement with the previous publication which showed that the typical composition of lignocellulosic biomass is approximately 41–45 wt% C, 5.7–5.9 wt% H, 1.2–1.8 wt% N and 0.2 wt% S (Osman et al. 2018). During the production of ACs and CNTs, the wt% of carbon significantly increased to approximately 70 wt%, which is in agreement with the EDX results shown in Table 1. Also, the method of synthesising CNTs from PK involved nitrogen-doping of the sample. Thus, an increase in nitrogen content of 7.4 wt% shown in Table 1 for that of CNTs is to be expected. EDX results revealed that 4 wt% of the CNTs surface is an iron species which is due to the presence of iron oxalate during the preparation of CNTs.

#### SEM-EDX analysis

**Fig. 3:**
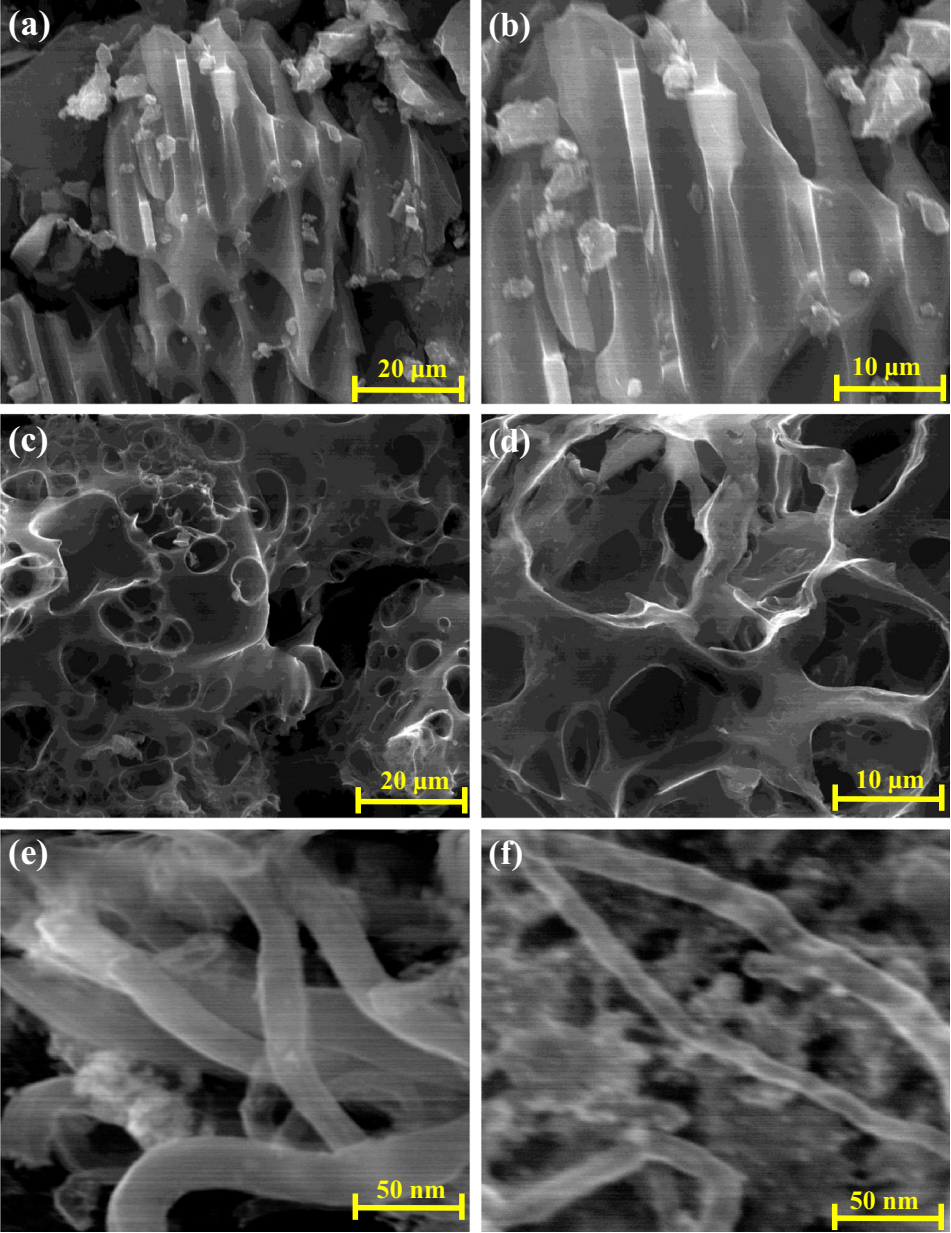
SEM images for potato peel ACs along with CNTs. **a**, **b** H_3_PO_4_ activation (PP), **c**, **d** KOH activation (PK) and **e**, **f** CNTs at a different level of magnifications using ETD detector

Figure 3 shows the produced ACs, along with the CNTs derived from PPW at different levels of magnification using the ETD detector. The PP sample showed porous or semi-porous carbonaceous structure as shown in Fig. 3a, while PK sample showed a better porous structure with formation of more channelling pores with multilayer formation as shown in Fig. 3b. This is in line with the S_BET_ results that showed an improvement in the surface area and the pore volume as shown in Table 1. The CNTs images in Figure 3c clearly showed carbon nanotubes, which is in agreement with the S_BET_ result that the expected formation of MWCNTs with the average number of walls present for the CNT is 40. The EDX results of the PPW, PP, PK and CNTs are shown in Table 1. The EDX analysis is a surface technique which gives the elemental composition in wt% which C is increased by 31.6 wt% from the PPW to the PP sample. This is maybe due to the breakdown of the lignocellulosic compounds and the evolution of the phenolic gases due to the decomposition of lignin and the formation of porous carbon materials. On the other hand, the 2.3 wt% of K in PPW sample disappeared through the formation of activated carbon (PP) as shown in Table 1. Phosphorus showed the opposite trend, where an increase in the wt% was observed between PPW and PP of 0.2 wt% and 2.2 wt%, respectively. This is maybe due to using phosphoric acid in the first activation method which disappeared in the second activation method (PK). The CNTs sample showed a 4 wt% of Fe, because of using iron oxalate during the preparation of CNTs from PK sample as shown in Table 1.

#### TGA/DTG analysis

**Fig. 4:**
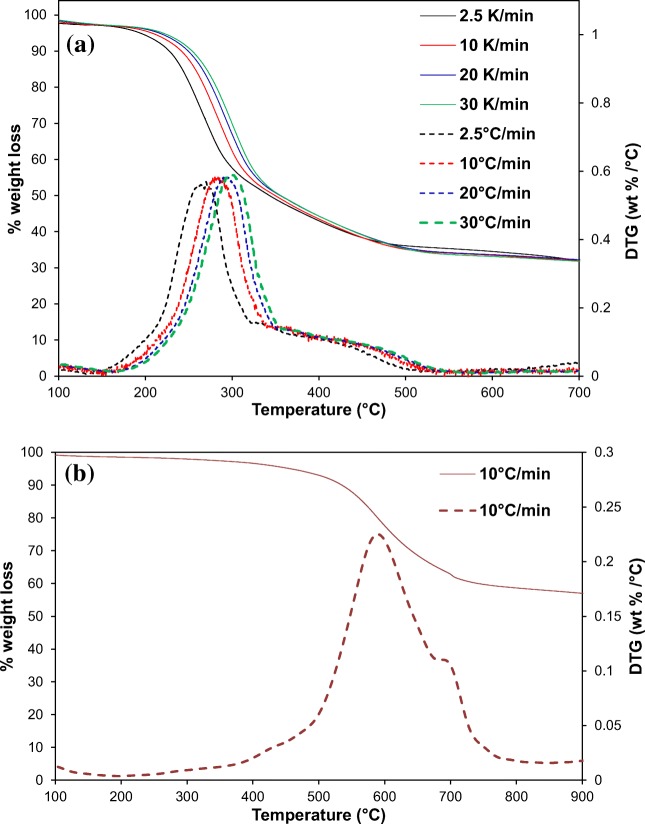
Thermogravimetric analysis of potato peel waste at different heating rates under nitrogen atmosphere, **a** TGA/DTG curves and **b** TGA/DTG curves of CNTs, where the TGA curves are solid lines and DTG are dashed lines

Thermogravimetric analysis (TGA) determines the thermal stability of a sample as shown in Fig. 4 for raw PPW and CNTs samples at multiple heating rates under a nitrogen atmosphere. The holocellulose components decompose easier than lignin in general; where hemicellulose and cellulose decompose in the temperature range of 200–375 °C and 275–380 °C, respectively, whereas lignin decomposes at a temperature range of 180–550 °C (Osman et al. 2017, Osman et al. 2018). Liang et al. reported that PPW showed a thermal decomposition that was in the temperature range of 200–500 °C, where herein, it was in the temperature range of 217–500^o^C (Liang &McDonald 2014). In general, there are four stages of thermal decomposition in biomass: at 50–130 °C (water evolution), 130–220 °C (degradation and evaporation of volatile compounds), 220–460 °C (depolymerisation pyrolysis) and > 460 °C (char formation) (Liang & McDonald 2014). They reported two distinct peaks in DTG results at 279 and 423 °C, where herein, a distinct peak was observed at 278 °C and a shoulder at heating peak of 425 °C (Liang &McDonald 2014). It is not surprising that the decomposition peaks shifted towards a higher temperature by increasing the heating rate, in this case, it involved increasing the heating rates from 2.5 to 30 °C min^−1^. The first decomposition peak clearly shifted by 52 °C. The TGA/DTG curves of the CNTs (Fig. 4b) showed 42 wt% of weight loss in the temperature range of 437–734 °C. This is in line with the work done by Abdolmaleki et al.(2015), who reported similar weight loss for multi-walled carbon nanotubes. From Fig. 4b, it can be determined that CNTs are much more thermally stable than the raw potato peel waste biomass.

#### TEM analysis

**Fig. 5:**
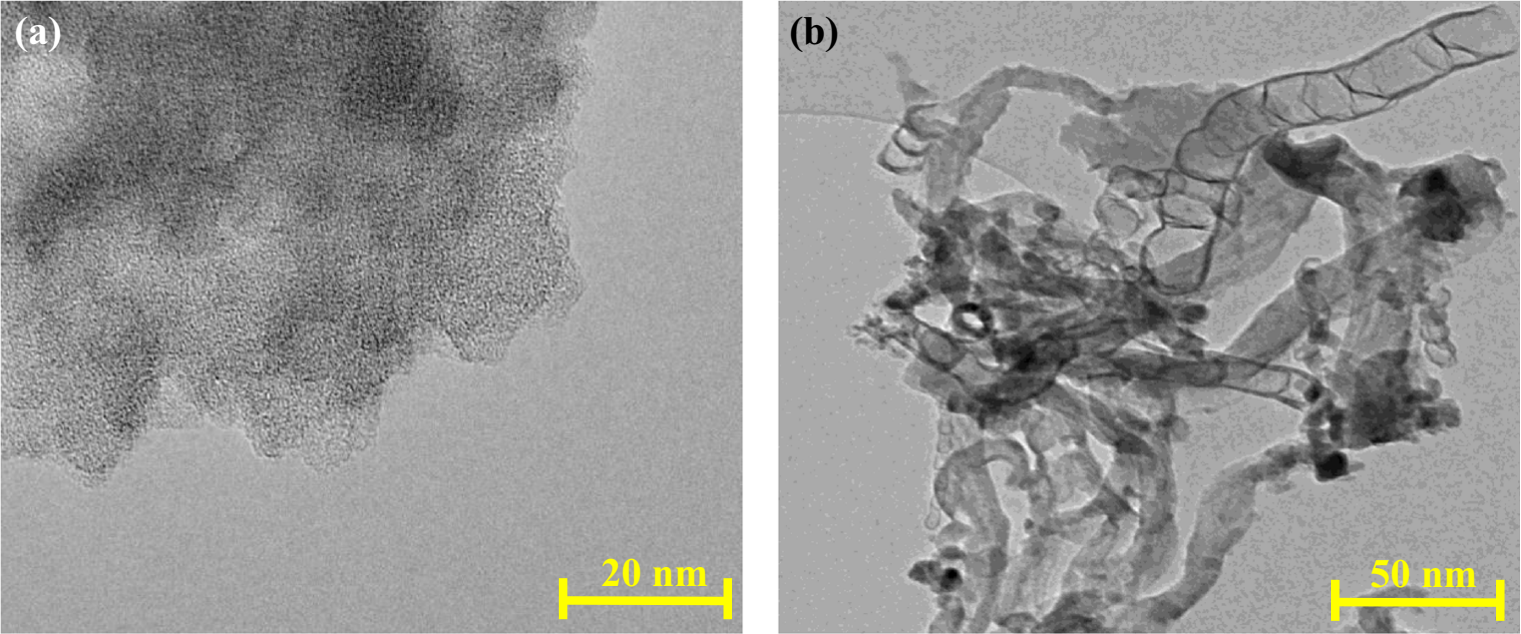
TEM images for **a** activated carbon (PK) and **b** CNTs

The TEM images of PK and CNTs are shown in Fig. 5a, b. The surface morphology of the PK is a porous multilayer texture, which is in agreement with the S_BET_ results (Fig. 2) that showed the formation of mesoporous carbonaceous material along with the SEM results in Fig. 3c, d. The CNTs sample in Fig. 5b showed the formation of carbon nanotubes, where the S_BET_ result of 52 m^2^ g^−1^ suggested multi-wall carbon nanotubes as there is a relationship between the surface area of the CNTs and the wall thickness.

#### XPS analysis

**Fig. 6:**
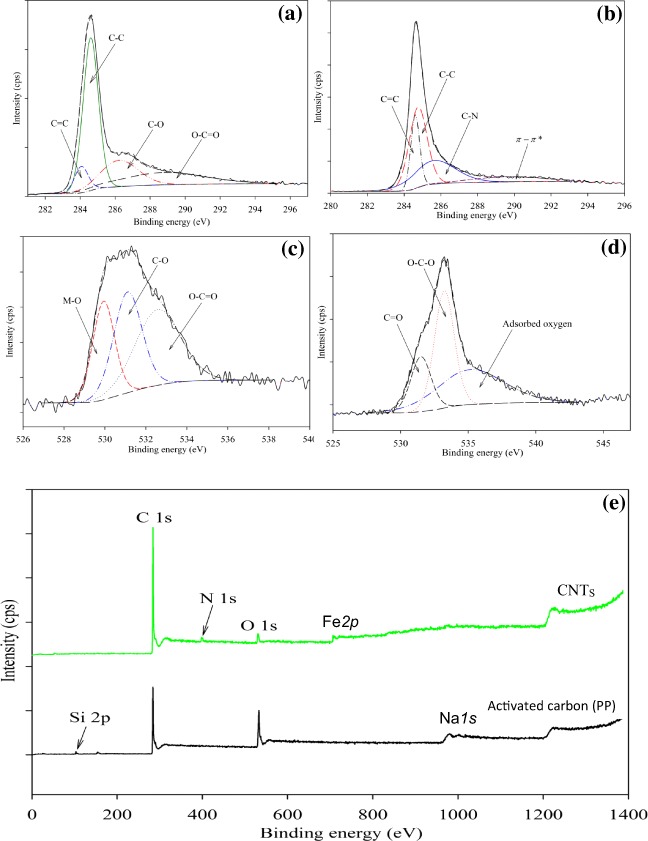
XPS of potato peel waste activated carbon **a** C*1s*, **b** C*1s*, **c** O*1s* and CNTs, **d** O*1s* along with **e** XPS survey

The XPS analysis of the activated carbon (PP) and the CNTs is shown in Fig. 6a–e, Figure S1 (supplementary) and Table 2. This was performed to detect the surface species composition and their binding energies, along with the oxidation states. The main C*1s* peak of PP and CNTs was observed at the binding energy of 284.7 eV, which is attributed to C-C bonding which is larger in PP than that of CNTs as shown in Fig. 6a, b. On the other hand, PP sample showed a peak at 284.1 eV (C=C) which significantly increased and shifted towards higher binding energy of 284.5 eV in the case of CNTs sample, implying the formation of strong carbon bonding within the CNTs structure. The PP sample showed two extra small peaks at 286.3 and 289.1 eV which are attributed to C-O and O-C=O bondings, respectively. While CNTs sample showed two extra small peaks at 285.5 eV (C-N) and 290.1 eV (π-π*) which is ascribed to the N-C=O in the N-doped carbon structure of the CNTs or C=O in the carbonyl group of the CNTs structure (Bhattacharjya et al. 2014). Table 2 showed that the wt% of carbon slightly increased from PP (89.4 wt%) to CNTs (90.2 wt%), nevertheless, O1s dramatically decreased from PP (8.3 wt%) to CNTs (4.3 wt%), inferring that the oxygen species diminished during the synthesis of CNTs and the oxide species shifted from C-O in the activated carbon (PP) structure to C=O in the CNTs structure as seen in Fig. 6c, d (Sadri et al. 2017). The dominant O1s peak in both samples (PP and CNTs) is C-O bonding at binding energy of 533.2 eV. The PP sample showed another peak at 529.7 eV which is attributed to the M-O bonding (where M=P). On the other hand, CNTs sample showed two extra peaks at 531.4 eV and 535.5 eV which are attributed to C=O and adsorbed oxygen species. The P*2p* peak significantly decreased from 1.3 wt% for PP to 0.2 wt% for CNTs as shown in Table 2 and Figure S1a. Furthermore, PP sample showed an XPS peak of *P2p*_***1/2***_ at a binding energy of 133 eV which is attributed to C-O-P bonding in the AC structure implying the presence of P^5+^ from the phosphoric acid used in the first activation process (Sofer et al. 2016). However, CNTs sample showed a small peak of P^0^ metal at a binding energy of 129.9 eV. The PP and CNTs samples showed the N*1s* at binding energies of 398.2 eV and 400.98 eV which are attributed to pyridinic N and pyrrolic N structures which are two of the three main constituents of nitrogen-doped *sp2* carbon material system. The dominant peak was for the pyridinic N, where nitrogen atom bonds with two carbon atoms at the edges or defects of CNTs and contributes one p electron to the π system (Cheng et al. 2013, Yadav & Dixit 2017) as shown in Figure S1c. The N*1S* peak tripled from 1.1 wt% for PP to 3.8 wt% for CNTs as shown in Table 2 and Fig. 6d. Moreover, a small peak of Fe*2p*_*3/2*_ appeared in the CNTs as a result of the presence of iron oxalate during the preparation of CNTs at a binding energy of 711.3 eV, which is attributed to γ-Fe_2_O_3_ phase (Figure S1d). The results are in line with the reported MWCNTs structures in the literature (Hyeon et al. 2001). The XPS survey (Fig. 6e) for both of PP and CNTs is similar apart from the appearance of an extra peak of Fe*2p* and the increasing of N*1s* peak along with the reduction of O*1s* during the transformation of activated carbon (PK) into CNTs.

**Table 2:** XPS results of the activated carbon PP along with the CNTs sample

Sample	C*1s*		O*1s*		N*1 s*		Si*2p*		P*2p*		Fe*2p*	
	Beak B.E.	Atomic wt%	Beak B.E.	Atomic wt%	Beak B.E.	Atomic wt%	Beak B.E.	Atomic wt%	Beak B.E.	Atomic wt%	Beak B.E.	Atomic wt%
PP	284.4	89.4	532.3	8.3	398.5	1.1	--	--	133.3	1.3	--	--
CNTs	284.4	90.2	531.1	4.3	398.6	3.8	101.2	0.3	129.3	0.2	706.9	1.1

#### The water contact angle

**Fig. 7:**
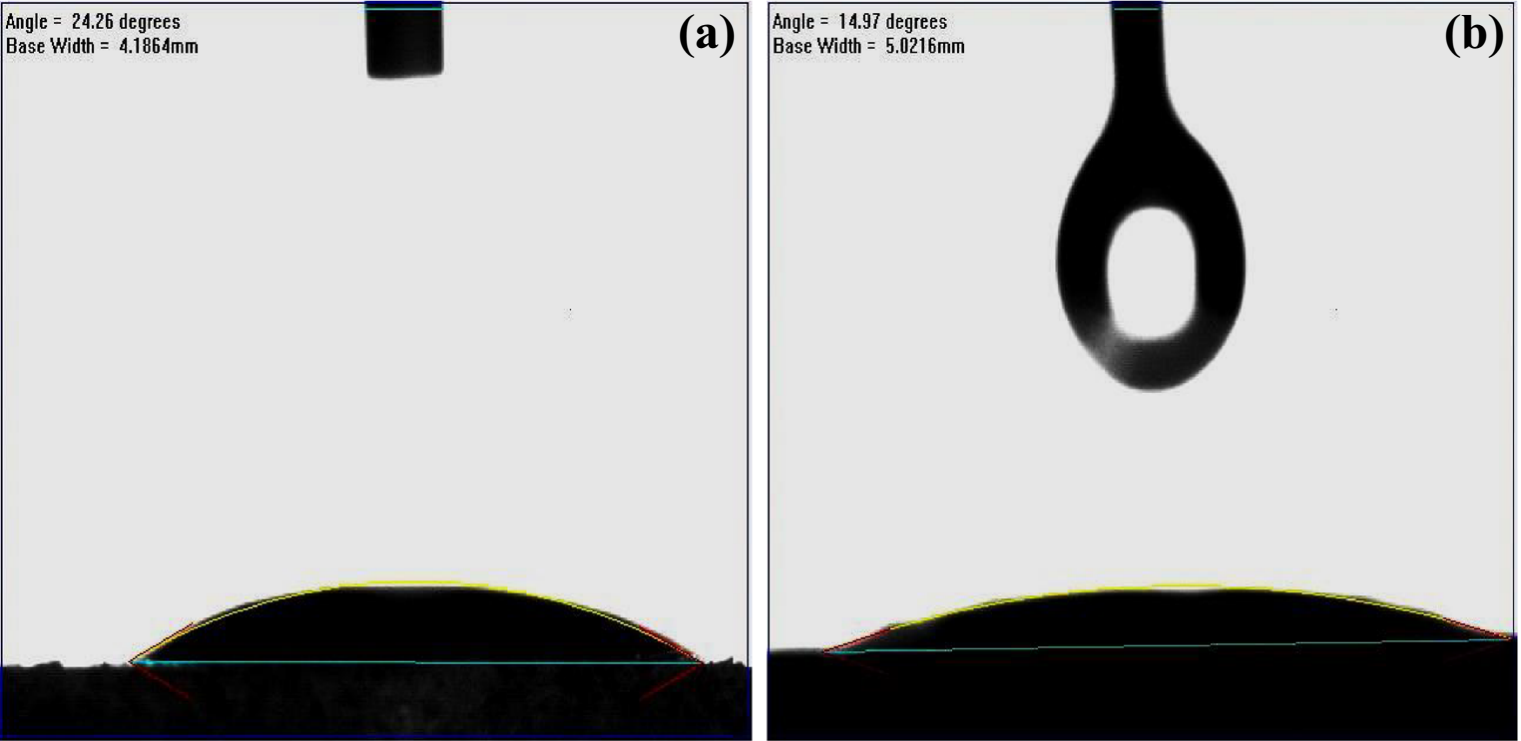
The water contact angle analysis of PPW (**a**) and the produced CNTs (**b**)

The water contact angle test gives an indication of the surface wettability, thus the test was performed on the raw PPW sample along with sample CNTs (Fig. 7a, b). The surface hydrophilicity is obtained as shown by Young’s equation (Eq. 1)

1 $$ \cos\ \uptheta =\frac{\gamma_{SV}-{\gamma}_{SL}}{\gamma_{LV}}\kern1em $$

where *γ*_*SV*_, *γ*_*SL*_ and *γ*_*LV*_ stand for the interfacial surface tension of solid (S), liquid (L) and gas vapour (V). According to the value of θ, surface wettability can be classified into four different categories: super-hydrophilic (θ < 10 °), hydrophilic (10 < θ < 90 °), hydrophobic (90 < θ < 150 °) and super-hydrophobic (θ > 150 °). The dry basis of the PPWwas hydrophilic as the water contact angle showed θ = 24.26 ° as shown in Fig. 7a. While the CNTs showed a lower contact angle than that of the PPW of θ = 14.97 °, indicating that the synthesised CNTs are more hydrophilic than the raw materials of potato peel waste as shown in Figure 7b. This is in agreement with the results published previously where CNTs showed contact angle in the range of < 15 ° (Janas & Stando 2017).

### Lead heavy metal removal results

**Fig. 8:**
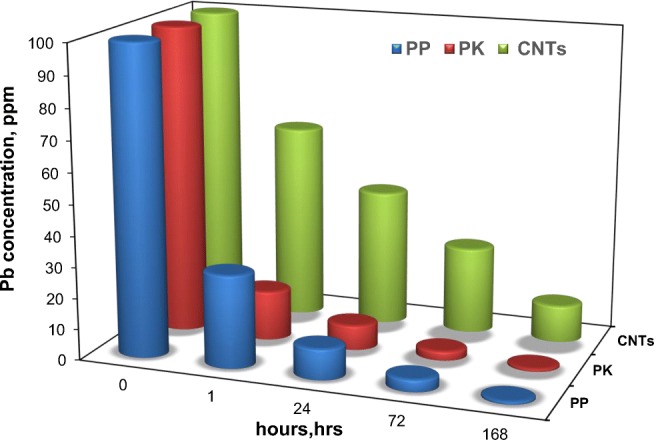
The heavy metal removal test of lead on PP, PK along with the CNTs over a period of 168 h

The produced materials have been tested in heavy metal removal (HMR) to determine how effective in the removal of lead (Pb^2+^) as a common heavy metal in wastewater (Acharya et al. 2009). The reason why lead was chosen for this study is that it is one of the most common heavy metals found in wastewaters. The industry requires its removal due to it being a systemic poison causing anaemia, kidney malfunction, tissue damage of the brain and even death in extreme poison (Acharya et al. 2009). Figure 8 showed the performance of ACs and CNTs in HMR from wastewater, where after 1 h of the test, the Pb^2+^ adsorption capacities were as follows: PK > PP > CNTs with adsorption values of 84, 70 and 37%, respectively. The PK sample showed the highest adsorption capacity due to its highest surface area along with the pore volume amongst the series of the samples herein as shown in Table 1 and Fig. 2. The effectiveness of this activated carbon may be due to the washing by hydrochloric acid where chloride can react with lead allowing for more effective removal of lead from the wastewater. It removed 92 and 97% of Pb^2+^ after 24 and 72 h of the test, respectively. The second most active material in HMR was PP where it showed slightly lower adsorption capacity than that of PK of 90 and 96% of Pb^2+^ after 24 and 72 h of the test, respectively. Although the CNTs showed the lowest removal rate based on a longer timeframe, it still removes 56 and 72% of Pb^2+^ over 24 and 72 h of the test, respectively, thus showing a high adsorption capacity. The lowest HMR of the CNTs may be due to the lower surface area along with the pore volume as shown in Table 1 and Fig. 2. Over 90% of the lead was removed by this activated carbon after just 1 day of testing.

The adsorption capacities of PP, PK and CNTs were calculated based on one initial concentration of Pb^2+^ and this were found to be 8.9, 9.3 and 5.7 mg g^−1^, respectively. Jones et al. (2016) used the modified *Dicerocaryum eriocarpum* mucilage as biosorption of heavy metals with biosorption capacity of 0.125 mg g^−1^. The adsorption capacity of biochar for heavy metal removal in Kolodynska’s work was around 6 mg g^−1^ (Kołodynska et al. 2012), while herein, it was 9.3 mg g^−1^ of activated carbon (PK). Wang et al. (2007) used MWCNTs in Pb^2+^ removal with adsorption capacity of < 4 mg g^−1^, while in this study, it was 5.7 mg g^−1^. On the other hand, adsorption capacity of Pb^2+^ removal using chitosan-modified fast pyrolysis biochar column adsorption was reported as 5.8 mg g^−1^ (Bombuwala Dewage et al. 2018). In a recent study, *Rhizopus oryzae* biomass was used to remove Pb^2+^ from wastewater with adsorption capacities ranging from 1.61 to 7.39 mg g^−1^ (Naeimi et al. 2018). Liu et al. (2019) used pure activated carbon in Pb^2+^ removal at pH = 6 with adsorption capacity of less than 10 mg g^−1^.

**Fig. 9:**
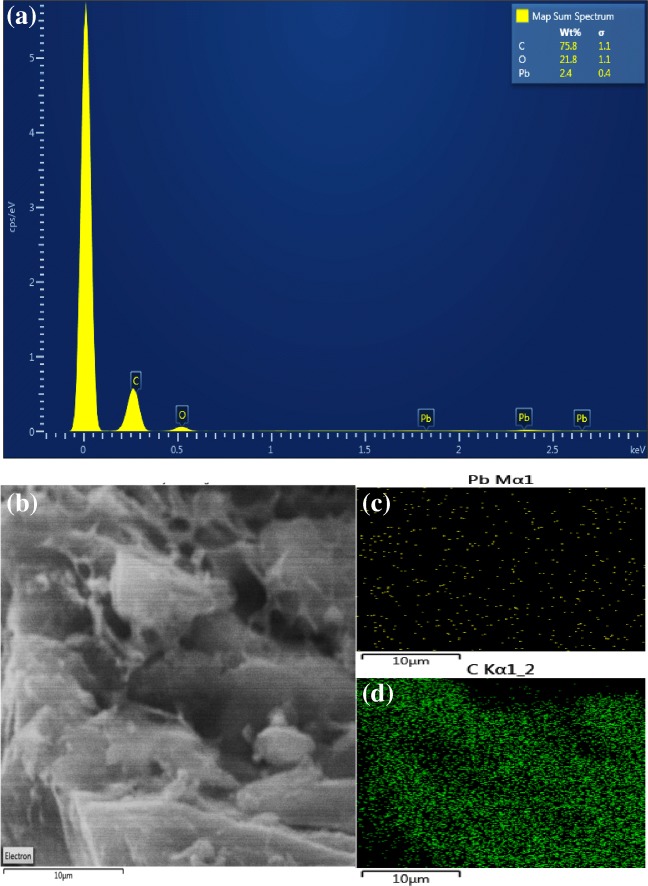
SEM-EDX analysis of potato peel waste on lead-activated carbon. **a** EDX results, **b** ETD image, **c** lead map and **d** carbon map

The most active material herein was investigated after the adsorption test (spent PK sample) to SEM/EDX. Figure 9 shows the EDX analysis along with the SEM image (ETD) and elemental mapping of Pb and C. The EDX result in Fig. 9a showed 2.4 wt% composition of the Pb in the spent PK sample, implying that some of Pb were adsorbed on the surface of PK during the HMR process. The rest of the composition were carbon and oxygen with 75.8 and 21.8 wt%, respectively, as shown in Figure 9a. The elemental mapping of Pb confirmed the presence of adsorbed lead as shown in Fig. 9c. In general, the elemental mapping showed that carbon is dominating the surface of the activated carbon (PK) compared with the lead metal.

## Conclusion

Herein, a waste type of lignocellulosic biomass was used for the production of activated carbon by means of two different activation steps. The first activation method using phosphoric acid (PP) created a porous-activated carbon with a surface area of 676 m^2^ g^−1^ and a pore volume of 0.26 cm^3^ g^−1^. This was a substantial increase and added value for the material compared with the parameters of the raw potato peel waste biomass which showed a very low surface area of < 4 m^2^ g^−1^. This activated carbon (PP) was further treated using a second activation step where potassium hydroxide as the activating agent was used (PK). This allowed a further increase in the surface area and pore volume which were found to be 833 m^2^ g^−1^ and 0.44 cm^3^ g^−1^, respectively. Finally, the CNTs that were produced using PK were found to be hydrophilic and be of the multi-walled type (MWCNTs). This was identified by the contact angle of θ = 14.97 °. Subsequently, the two activated carbon samples and the CNTs produced were tested in the application of heavy metal removal (HMR) with the potential to remove up to 84% of Pb^2+^ within the first hour of operation. Of these materials tested, PK showed the highest adsorption capacity in HMR, making it the ideal candidate in the rapid removal of heavy metals in wastewater treatment or in alternative adsorption applications. Our approach outlined to high surface area ACs and hydrophilic MWCNTs from this particular waste stream helps address and apply the circular economy concept by up-cycling an otherwise waste feedstock by adding value and other potential routes for application such as wastewater treatment and other end uses for AC and CNTs. Our approach opens doors for designing an environmentally sustainable ACs and CNTs from potato peel waste material which could be of great interest for many industrial applications, including wastewater remediation application along with microelectronics, smart and novel composites and bone growth in tissue engineering. It is anticipated that such an approach will make the synthesis of such value-added materials more sustainable, more eco-friendly and less expensive.

## Electronic supplementary material

ESM 1 (DOCX 169 kb)
