# Supplementary material for: Production and characterisation of activated carbon and carbon nanotubes from potato peel waste and their application in heavy metal removal
Source: Environ Sci Pollut Res Int. 2019 Nov 20;26(36):37228–41. doi: 10.1007/s11356-019-06594-w (PMC6937222; doi:10.1007/s11356-019-06594-w)
Supplement: Supplementary file 1 — (DOCX 169 kb) [file 11356_2019_6594_MOESM1_ESM.docx]

***Supplementary information of***

**Production and characterization of activated carbon and carbon nanotubes from potato peel waste and their application in heavy metal removal**.

**Ahmed I. Osman ^a,b*^, Jacob Blewitt ^a^, Jehad K. Abu-Dahrieh ^a^, Charlie Farrell ^c,d^, Ala'a H. Al-Muhtaseb ^e^, John Harrison ^c^, David W. Rooney ^a*^**

^a^ School of Chemistry and Chemical Engineering, Queen’s University Belfast, Belfast BT9 5AG, Northern Ireland, UK.

^b^ Chemistry Department, Faculty of Science - Qena, South Valley University, Qena 83523 – Egypt.

^c^ South West College, Cookstown, Co. Tyrone, BT80 8DN, Northern Ireland, UK.

^d^ School of Mechanical and Aerospace Engineering, Queen’s University Belfast, Belfast BT9 5AH, Northern Ireland, UK.

^e^ Department of Petroleum and Chemical Engineering, College of Engineering, Sultan Qaboos University, Muscat, Oman.

Corresponding Author: David Rooney, Ahmed Osman

Email:d.rooney@qub.ac.uk, [aosmanahmed01@qub.ac.uk](mailto:aosmanahmed01@qub.ac.uk)

Address: School of Chemistry and Chemical Engineering, Queen's University Belfast, David Keir Building, Stranmillis Road, Belfast BT9 5AG, Northern Ireland, United Kingdom

Fax: +44 2890 97 4687

Tel.: +44 2890 97 4412


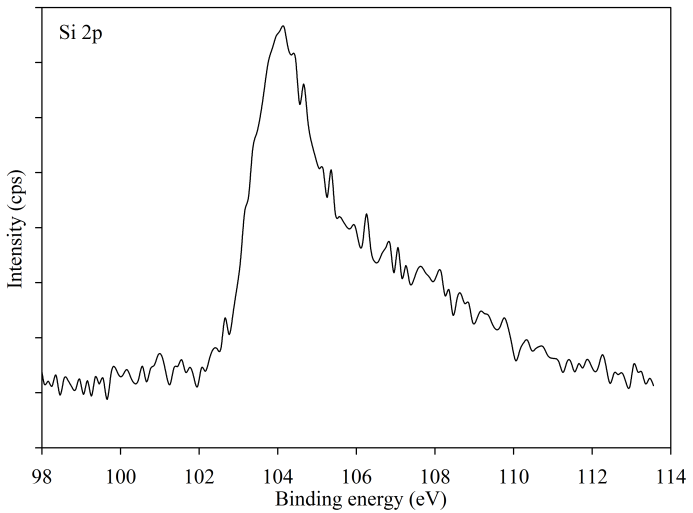


Activated carbon (PP)

CNTs

b)

a)


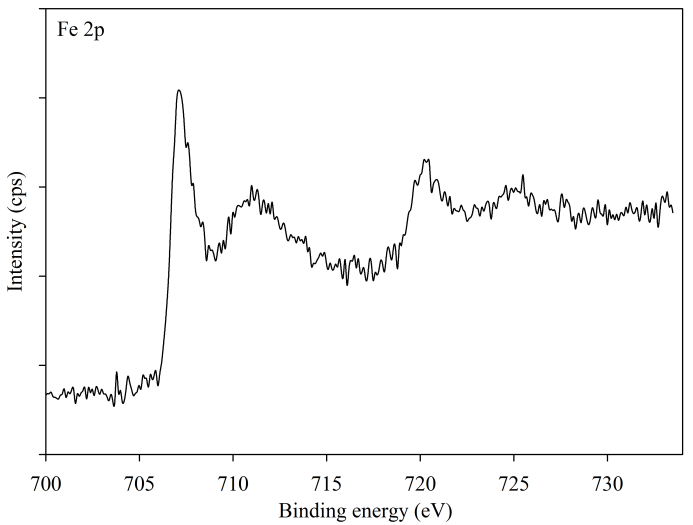

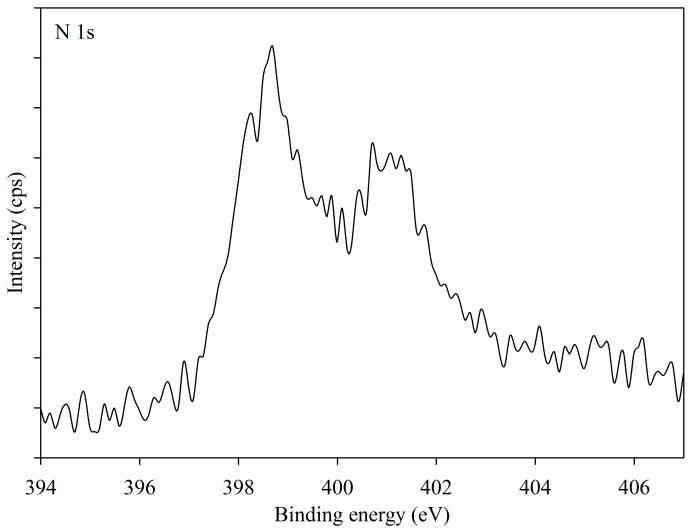


**pyridinic N**

γ-Fe_2_O_3_

d)

c)

**pyrolic N**

(b)

**Figure S1:** High resolution spectra of a) P*2p* for both activated carbon (PP) and CNTs, b) Si*2p* peak for PP sample surface, c) N*1s* peak for CNTs sample surface and d) Fe*2p* peak for CNT_S_ sample surface.
